# Shall We Dance? Dancing Modulates Executive Functions and Spatial Memory

**DOI:** 10.3390/ijerph17061960

**Published:** 2020-03-17

**Authors:** Carmen Noguera, Dolores Carmona, Adrián Rueda, Rubén Fernández, José Manuel Cimadevilla

**Affiliations:** 1 Department of Psychology, University of Almeria, 04120 Almería, Spain; cnoguera@ual.es (C.N.); lola.cg@hotmail.com (D.C.); drilan@gmail.com (A.R.); 2 Health Research Center, University of Almeria, 04120 Almería, Spain; rubenfer@ual.es; 3 Department of Nursing, Physiotherapy and Medicine, University of Almeria, 04120 Almería, Spain

**Keywords:** virtual reality, aging, sport, hippocampus, neuropsychology

## Abstract

Background: Aging is generally considered to be related to physical and cognitive decline. This is especially prominent in the frontal and parietal lobes, underlying executive functions and spatial memory, respectively. This process could be successfully mitigated in certain ways, such as through the practice of aerobic sports. With regard to this, dancing integrates physical exercise with music and involves retrieval of complex sequences of steps and movements creating choreographies. Methods: In this study, we compared 26 non-professional salsa dancers (mean age 55.3 years, age-range 49–70 years) with 20 non-dancers (mean age 57.6 years, age-range 49–70 years) by assessing two variables: their executive functions and spatial memory performance. Results: results showed that dancers scored better that non-dancers in our tests, outperforming controls in executive functions-related tasks. Groups did not differ in spatial memory performance. Conclusions: This work suggests that dancing can be a valid way of slowing down the natural age-related cognitive decline. A major limitation of this study is the lack of fitness assessment in both groups. In addition, since dancing combines multiple factors like social contact, aerobic exercise, cognitive work with rhythms, and music, it is difficult to determine the weight of each variable.

## 1. Introduction

Aging is considered in some studies as one of the most prominent problems in the short and medium term for modern societies [1], demanding more support and investment in health systems. It is widely accepted that normal aging is associated with a decline in brain functions [2], disturbing, among others, executive and memory processes [3].

Spatial memory is reported as one of the most affected abilities by normal and pathological aging, mainly due to the decline of medial temporal lobe-dependent functionality [4]. This cognitive function allows us encoding, storing and retrieving information about spatial locations and stimuli [5].

Until recent years, the assessment of spatial memory skills in humans was inaccurate due to the lack of ecologically valid tests. This issue was solved thanks to technological advancements, which enabled the development of tests that could recreate varied and realistic environments in a more ecological way [6,7,8,9,10]. In this regard, virtual reality based tests allowed to find differences that traditional spatial measurements were unable to report [11,12]. Some tests in this paradigm also proved capable of predicting mild cognitive impairment where traditional measures failed [13].

In addition to spatial memory, executive processes such as inhibition, working memory, and cognitive flexibility, are also disturbed by age-related changes [14]. All these top-down mental processes are needed for mental and physical health, since they allow us to face new situations, solve problems, control our behavior, keep a goal in mind or concentrate and pay attention to perform a task [15]. The lack of sleep and/or exercise, loneliness, or stress, often present in our daily lives, can also each impair these executive functions.

At the experimental level, it is feasible to evaluate the executive component through the so-called ANT or Attentional Network Test [16]. Originally, Fan, Posner and collaborators [16] developed a procedure that combined the flanker task [17] with the visual cueing paradigm [18], to allow testing the efficiency of three attentional networks (the orienting, the alerting and the executive control networks) proposed by Posner and Petersen [19,20]. The traditional version and subsequent modifications of this task have showed their independence, but also the cooperation between the three networks [21,22,23]. This procedure has been widely used to assess the components of visual attention in healthy adult [16,21,22], child population [24], and subclinical persons [25,26], but also in a diversity of studies including genetic studies [27] and neuroimaging [28], or meditation [29].

More recently, a version of the ANT task called ANT-I task (Attentional Network Test-Interactions) adapted by Callejas et al. [21], that combines the flanker task with the auditory (instead of visual) cueing paradigm, was applied to study the effect of acute aerobic exercise on cognitive functions. Namely, Huertas and his colleagues [30] found accelerated reaction times and a reduced alerting effect (compared with the rest condition) in a highly experienced cyclists group. However, this kind of exercise did not modulate the functioning of either the orienting or the executive control attentional networks [30]. In another recent study, Noguera and her collaborators assessed both the spatial memory and the functioning of the three attentional networks in a sample of men distributed according to their age (60–69 and 70–79 years old), and aerobic exercise practice (sedentary vs sportsmen). Overall, sportsmen outperformed sedentary participants in most of the measures employed, showing a better spatial orienting and a more effectively functioning of the three networks [31].

Therefore, there are some factors that could help to slow down or even reverse the negative effects of normal aging. Namely, the practice of physical activity has been reported to improve spatial memory and executive functions of adults and elders [31,32,33,34]. This is connected with the increases of both grey and white matter resulting in better cognitive abilities [35,36]. It is hypothesized that aerobic sports ultimately contribute to increase the cognitive reserve which is considered a neuroprotective factor from cognitive decline [37].

Just as running, swimming or walking are considered type of aerobic exercise, most forms of dance may be also considered an aerobic exercise, and as such could contribute to reduce the risk of cardiovascular disease, help weight control, or to modulate the stress and depressive symptoms [38]. Dancing also involves other skills like coordination with music, and retrieval of sequences of movements, reinforcing spatial perception and memory [39] as well as executive functions [40]. It was demonstrated that elders practicing dancing activities reported remarkable improvements in equilibrium and consistency of steps [41]. These authors also showed that dancing is more beneficial than repetitive physical exercises, thus activating brain plasticity mechanisms at a greater extent. Pronounced differences in brain volumes were also found after an exhaustive dancing training [42]. However, those studies were unable to find differences in spatial memory due to the use of less sensitive tasks. On the contrary, using the virtual reality paradigm, which consistently proved to be superior to traditional spatial measurements, may be able to succeed in this matter [11,12,13]. 

Thus, it would be interesting to explore whether the practice of another type of aerobic exercise, such as salsa dancing, could have some beneficial effects for cognitive health.

The aim of this study was to assess if dancing, that includes lots of jumping or turning, affects spatial memory and executive functions using more sensible virtual reality tasks like virtual reality-based tasks and ANT-I task for assessing spatial memory and executive functions, respectively. Given the beneficial effect that aerobic exercise has on our mental health, we hypothesized that dancers will show better performance than no-dancers.

## 2. Materials and Methods

### 2.1. Participants

A sample of 46 healthy adults participated in this study (aged 49–70 years). Twenty-six were dancers (14 men, 12 women) and twenty control participants (10 men, 10 women). Participants were recruited at dancing academies, old-adults university courses and social clubs in Almeria. An interview was made in order to obtain information about their sport habits, videogames practice, health condition and academic background (see Table 1). Those with any neurological disease, traumatic brain injury, cardiopathy, drug intake, or any other condition that could interfere with performance were excluded. To be classified as dancers, participants must have been practicing salsa dancing for at least six months prior to the study and did not practice any other kind of aerobic activity during the last year. In contrast, control participants had not practiced dancing or any sport for the same period of time. All participants were recruited from the province of Almeria. The study was approved by the University of Almeria Ethical Committee (UALBIO2015/012) and fulfills the requirements of the European Communities Council Directive 2001/20/EC and the Helsinki Declaration for biomedical research involving humans. Participants were informed in advance that they would be included in a study examining spatial memory and executive functions, though the hypotheses of the study were never revealed. They were also told that they were free to leave the experiment at any time.

### 2.2. Materials

The following neuropsychological tests were applied: (1) the Kaufman Brief Intelligence Test (K-BIT, [43]). (2) The FAS Word Fluency Test [44] to measure phonologic and semantic fluency. (3) The Zoo Map subtest of the BADS battery (Behavioral Assessment of Dysexecutive Syndrome, [45]) to assess planning ability.

In addition, we used an adapted version of the ANT-I task [21] to evaluate the ability of participants to prepare to response faster in the presence of a warning signal to orient their attention according to the information provide by a spatial cue (valid vs invalid), and to efficiently respond to a target flanked by distractors.

Finally, to assess spatial ability, the Boxes Room task was used, which consisted in a virtual room where participants had to find 3 rewarded boxes out of 16 possible rewarded positions in ten consecutive trials (see [8]). They used a joystick to move around the room. The task was administered on an HP 2300-MHZz notebook equipped with 6000 MB of RAM and a 15 inch XGA TFT color screen (1024 × 768).

### 2.3. Procedure

The evaluation took place in the Neurosciences laboratories at the University of Almeria. All participants were tested individually. Participants received verbal and written instructions before each test and they were administered in the following order: Interview, K-BIT, Zoo Map test, FAS, ANT-I task, and Boxes Room task. Assessment lasted between 1 h 30 and 2 h, taking a break after completion of each one.

Kaufman brief intelligence test. This test consists of two subtests. In the verbal subtest, participants observed a series of pictures and named the object represented on them. For the second part of this subtest, a definition, together with some letters of the name were given as cues, and participants had to say the name that the definition referred to. For the non-verbal part, participants had to find out the relationship in a set of meaningful (people or objects) and abstract stimuli (symbols) and point to the correct answer among all the ones given.

The FAS Word Fluency Test. In this test, participants were required to name as many words as possible in one minute starting with the letters F, A, and S, excluding proper nouns and diminutives (phonemic verbal fluency). For the categorical fluency part, they were asked to generate animal names (Animal Naming) as far as possible in one minute, having no restrictions during the stage.

The Zoo Map Test. Participants observed the map of a zoo and they were instructed to draw a line from the entrance (starting point) to the Cafeteria (finish point) with some limitations. Thus, it was mandatory to go through a number of locations before they reached the finish point. Besides, some roads could only be taken once, while others could be taken as many times as they wanted.

**Figure 1:**
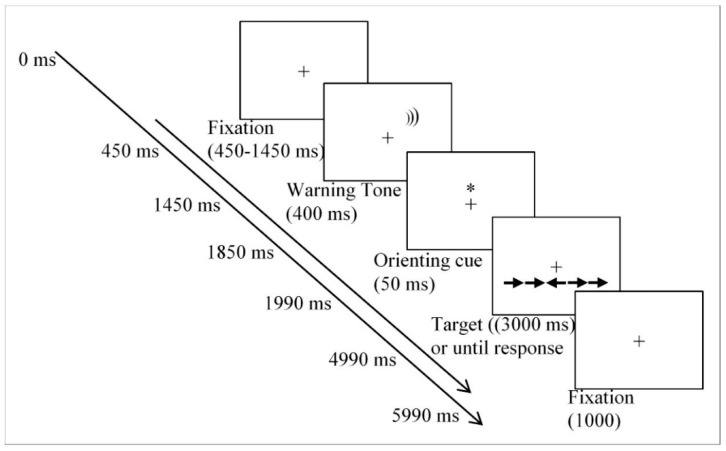
Example of procedure in the ANT-I (Attentional Network Test-Interactions). The timing of events is presented on the left.

The ANT-I Task. Participants had to respond to the direction of a central arrow, as fast and as accurately as possible, by pressing the “C” key with the left hand if a leftward arrow was displayed, and the “M” key with the right hand if that rightward arrow appeared. Figure 1 illustrates the sequence of events presented in each trial. Firstly, a central plus sign was presented for 450–1450 ms. Secondly, a 2000 Hz alerting sound appeared in half of the trials for 400 ms. This warning signal only indicated that participants should be prepared to respond to the upcoming target. Thirdly, a spatial orienting cue (an asterisk) was presented for 50 ms above or below the plus sign on 2/3 of the trials, specifying information on where the target will appear. No cues appeared in the remaining trials. Finally, the target and flankers were presented either on the same or opposite locations of the previous orienting cue for 3000 ms or until participants’ response. The fixation point was again presented for 1000 ms, before starting the next trial. The presence or absence of the alerting sound (tone) constituted the two levels of alerting condition. The orienting variable was defined by the presence of the spatial cue (valid trials, when the cue was presented at the same location as the target arrow; invalid trials, when the spatial cue appeared at the opposite location to the target), or the absence of the spatial cue (no-cue trials, when the cue was not presented). The conflict condition was made by means of flankers, consisting of four arrows identical to the target, surrounding the target either pointing in the same direction as the target (congruent condition), or pointing opposite direction (incongruent condition).

Participants performed a practice block of 12 trials, followed by 4 experimental blocks of 48 trials each, 24 trials per each alerting (tone/no tone) and executive condition (congruent/incongruent), and 24 trials per orienting condition (16 trials with spatial cue and 8 trial with no cue. The trials were randomly ordered within each block.

The Boxes Room Task. This task, described in [8], consisted of a square virtual room with sixteen brown boxes, distributed in 4 rows with 4 boxes per row. The room also displayed different elements located on the walls to disambiguate the room, like a door, a window and several paintings. Three out of the sixteen boxes were rewarded, and participants were asked to open the boxes one by one in order to find them. To do this, they used a joystick to navigate through the environment. To open a box, they must be situated in front of it and press a button. If the box was rewarded, it changed its color from brown to green and a pleasant melody sounded. In contrast, if the box opened was incorrect, it turned to red color and an unpleasant melody sounded. They were instructed to open the lowest number of incorrect boxes while opening the rewarded ones, and to do this as fast as possible. The task was composed of ten trials. Rewarded boxes were located in the same position during the ten trials. Each trial ended when the participants opened all the rewarded boxes or after 150 s elapsed. Number of errors committed were registered.

### 2.4. Statistical Analysis

Kolmogorov–Smirnov test was used to estimate normality. When the normality assumption was not met, samples were compared with a Mann–Whitney U test for independent samples. Non-parametric tests were required for analyzing performance in the Zoo map task.

A two-way ANOVA (Group (Dancers vs. Control) × Gender) was applied to analyze the scores of K-BIT and FAS-A tasks. Errors in the Boxes Room Task were analyzed using an ANOVA (Group (Dancers vs. Control) × Gender × Trial) with repeated measures in the last variable. Fisher’s Least Significant Differences (LSD) test was applied for post-hoc analyses.

Regarding ANT-I task, trials containing an incorrect response (1.3% of trials), or those with reaction times falling more than 2.5 standard deviations from the overall mean RT (1.7% of trials), were removed from analyses. Mean RTs and errors were analyzed employing a mixed design ANOVA with Group (Dancers vs Control) and Gender as the between-subjects factors, and Auditory Signal (Tone, No Tone), Spatial Cue (No Cue, Valid, Invalid), and Flanker Type (Congruent, Incongruent), as the within-subjects factors. The alerting effect was calculated as the difference between the trials with auditory signal trials and those without it. The orienting effect was obtained by calculating the difference between the valid and invalid trials. The conflict effect was calculated as the difference between the congruent and incongruent trials.

Finally, a Pearson correlation was estimated between Age and the set of variables used in this study. An ANCOVA was run to determine the effect on those statistically significant correlations. Differences were considered significant if *p* < 0.05. The statistical analyses were performed using STATISTICA, version 13 (TIBCO, Palo Alto, CA, USA) and IBM SPSS, version 25 (SPSS Inc., Chicago, IL, USA).

## 3. Results

Data fulfilled the assumption of normality in all the variables studied but Zoo test. In addition, a Pearson correlation showed a statistically significant negative correlation only between Age and naming tests (Fas-A and Animal Naming) (see results in point 3.3).

### 3.1. K-BIT (General Intelligence Measurement)

**Figure 2:**
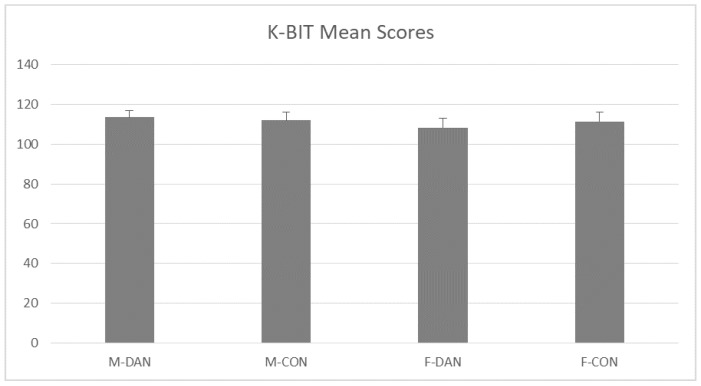
Kaufman Brief Intelligence Test (K-BIT) mean scores. Groups were very homogeneous in intelligence. No group differences emerged. Male dancing (M-DAN); female dancing (F-DAN); male control (M-CON); female control (F-CON). Mean + SEM.

ANOVA showed that groups were very homogeneous. Neither the Gender factor (F(1,43) = 0.455 *p* = 0.51), Group (F(1,43) = 0.042 *p* = 0.83), nor the interaction Gender x Group (F(1,43) = 0.58 *p* = 0.58), produced statistically significant effects (see Figure 2).

### 3.2. Zoo Test

**Figure 3:**
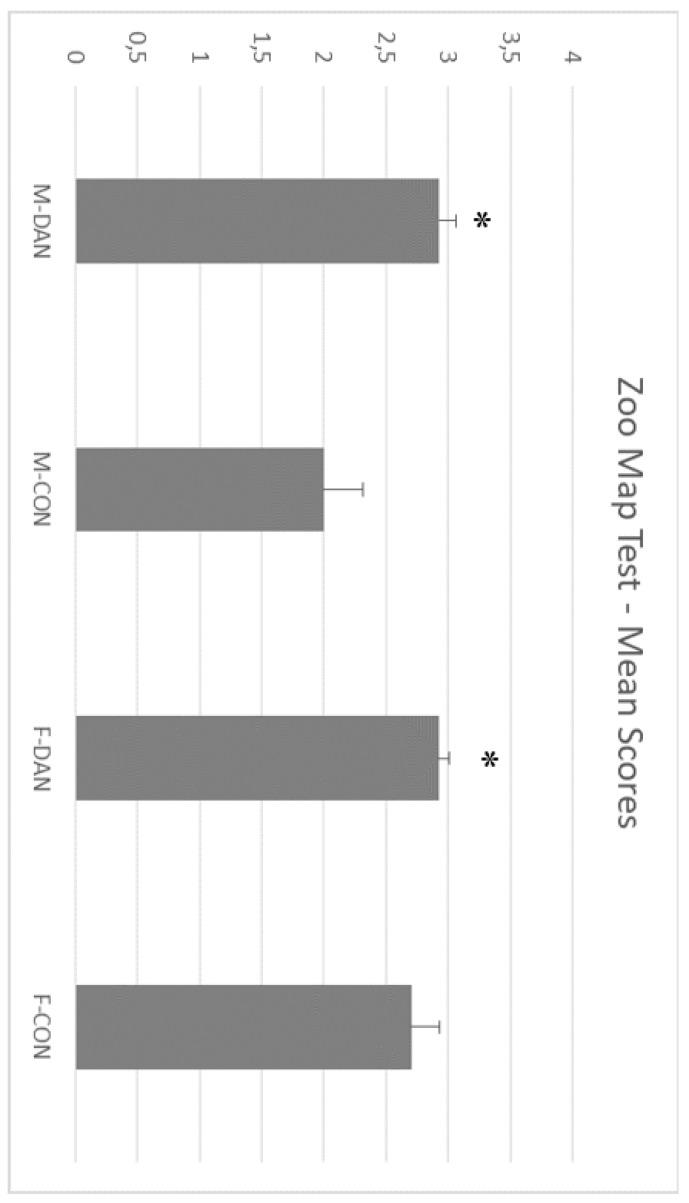
Zoo map test. Note that dancing groups outperformed controls. This indicates that they are more efficient in planning. Male dancing (M-DAN); female dancing (F-DAN); male control (M-CON); female control. Mean + SEM. * Significant differences with the control counterpart (*p* < 0.005).

Mann–Whitney U test for independent samples disclosed significant differences between dancers (mean range 27.79) and controls (mean range 17.93) (U = 148.5, *p* < 0.005) (see Figure 3).

### 3.3. FAS-A Test and Animal Naming

**Figure 4:**
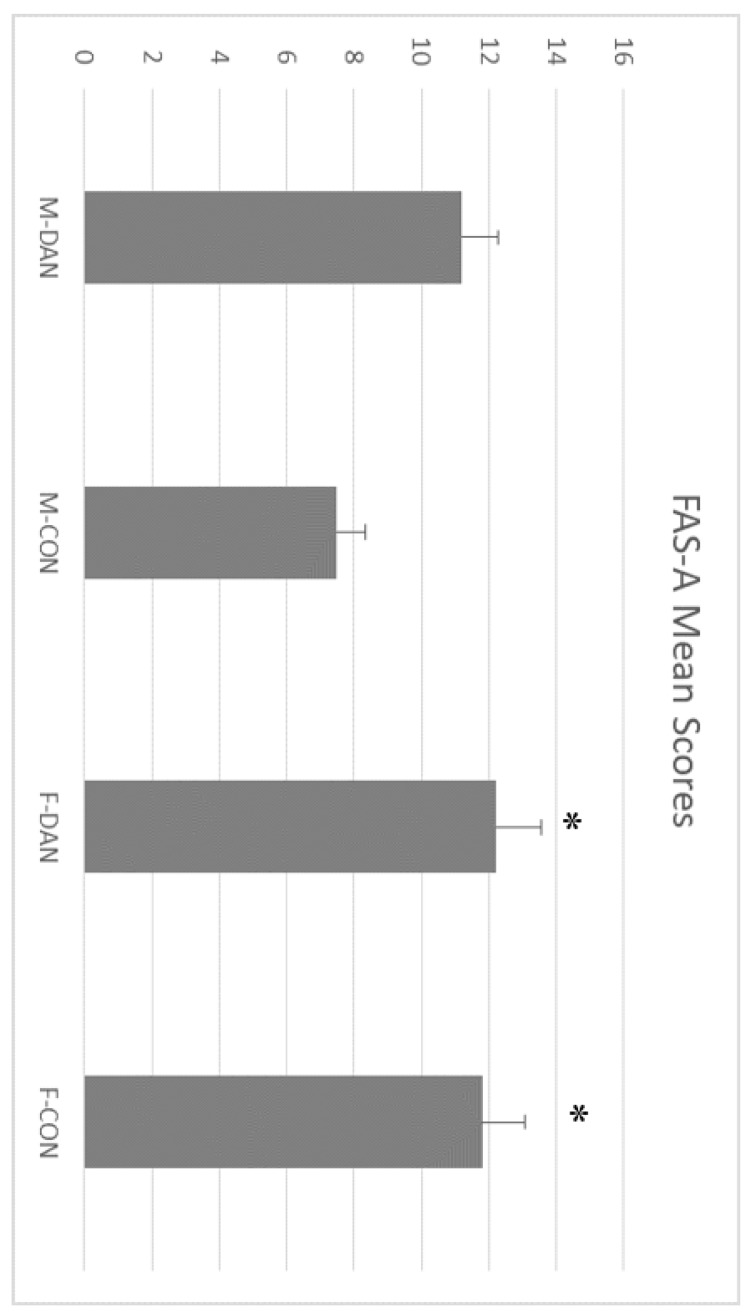
FAS Word Fluency Test mean scores. Women outperformed men in this task. This was independent of the practice of dancing. Male dancing (M-DAN); female dancing (F-DAN); male control (M-CON); female control (F-CON). Mean + SEM. * Significant differences with men (*p* < 0.05).

ANOVA applied to FAS-A test showed a significant main effect of Gender factor, F(1,43) = 5.47; *p* = 0.020), but no effect of Group factor, F(1,43) = 3.33; *p* = 0.070), or Gender x Group interaction, F(1,43) = 2.09; *p* = 0.150). Mean scores showed that women ($\bar{X}$ = 12.03) outperformed men ($\bar{X}$ = 9.66) in this task, although this was not linked with the practice of dancing (see Figure 4). In addition, a Pearson correlation showed a statistically significant negative correlation between Age and Fas-A (−0.455, *p* < 0.001) and Age and Animal Naming (−0.563, *p* < 0.001). ANCOVA confirmed the significant effect of Age on both test; Fas-A (t = −3.16, *p* < 0.003) and Animal Naming test (t = 4.29, *p* < 0.001).

**Figure 5:**
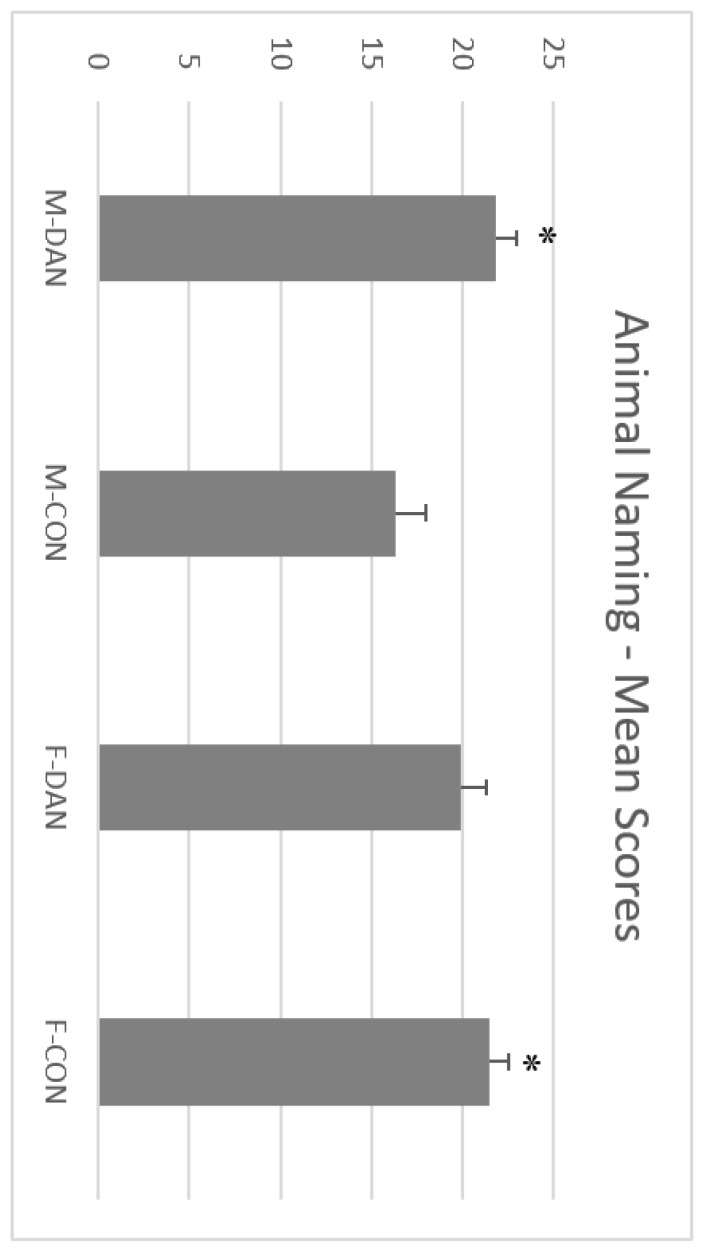
ANIMAL NAMING. Female controls and men in the dancing group scored better than men controls. Male dancing (M-DAN); female dancing (F-DAN); male control (M-CON); female control (F-CON). Mean + SEM. * Significant differences with men controls (*p* < 0.05).

Scores of Animal Naming were also analyzed. ANOVA revealed that there were neither a significant main effect of Gender, F(1,43) = 1.72; *p* = 0.190), nor Group, F(1,43) = 2.56; *p* = 0.110), but a statistically significant interaction Gender x Group, F(1,43) = 8.23; *p* = 0.006). A post-hoc analysis (Fisher LSD Post-hoc test) showed that men in the dancing group ($\bar{X}$ = 21.85) and women in the control group ($\bar{X}$= 21.50) scored better than men in the control group ($\bar{X}$ = 16.30) (see Figure 5).

### 3.4. The ANT-I Task

#### 3.4.1. Latencies

Data from the analysis of variance (see Table 2) showed revealed a significant main effect of Group, F(1,42) = 4.07; *p* = 0.05; ηp2 = 0.08, because of dancers responded faster (664.86 ms) than sedentary group (717.30 ms). The Gender main effect was not significant, F(1,42) = 0.03; *p* = 0.96. However we observed a significant interaction between Group and Gender, F(1, 42) = 5.68; *p* = 0.02; ηp2 = 0.12, due to that men dancers were fasters (635 ms) than controls (749 ms), F(1,22) = 6.01; *p* = 0.023; ηp2 = 0.21, while no differences were found between the two groups of women (695 ms for dancers vs 686 ms for no dancers), F(1,20) = 0.24; *p* = 0.62. Men dancers also responded faster (635 ms) than women dancers (695 ms), F(1,24) = 7.75; *p* = 0.01; ηp2 = 0.24. However, the difference between the groups of no dancers (men = 749 ms; women = 686 ms) was not significant, F(1, 18) = 1.46; *p* = 0.24).

We also found main effects of Auditory Signal, F(1,42) = 81.69; *p* = 0.000; ηp2 = 0.66, due to faster responses in the tone (678.54 ms) than those in the non-tone trials (703.61 ms); Spatial Cue, F(2, 84) = 51.82; *p* = 0.000; ηp2 = 0.55 (invalid = 708.08 ms; valid = 668.71 ms; no cue = 696.44 ms); and Flanker Type, F(1,42) = 804.11; *p* = 0.000; ηp2 = 0.95 (Congruent = 636.08 ms; Incongruent: 746.08 ms). We observed an interaction between Auditory Signal and Spatial Cue factors, F(2,84) = 8.43; *p* = 0.000; ηp2 = 0.17, indicating a significant larger orienting effect in the tone (46 ms) than in the no-tone trials (33 ms), F(1,45) = 4.23; *p* = 0.040; ηp2 = 0.09. In addition, a significant Auditory Signal x Flanker Type interaction was observed, F(1,42) = 7.60; *p* = 0.008; ηp2 = 0.15, since the presence of a tone improved the response speed in both congruent (619.8 ms) and incongruent trials (737.2 ms), compared to that of trials without tone (congruent 652.3 ms vs. incongruent 754.9 ms), with a conflict effect of 117 ms in the tone condition, relative to that of 103 ms observed in the non-tone condition. A new ANOVA was applied in each group to disclose their patterns of performance in the task.

Dancing group. The analysis showed a significant main effect of Auditory Signal, F(1,25) = 45,04; *p* = 0.000; ηp2 = 0.640, due to lower latency in the tone condition (649.33 ms) than in the non-tone condition (675.84 ms). The alerting effect was of 26.61 ms. There were also a significant main effect of Spatial Cue, F(2,50) = 32.63; *p* = 0.000; ηp2 = 0.570, since participants responded more quickly in the valid trials (642.44 ms) than in the invalid (679.45 ms) or no cue trial (665.72). The orienting effect was of 37.01 ms. The Flanker Type was also statistically significant, F(1,25) = 482.30; *p* = 0.000; ηp2 = 0.950, because of faster responses in the congruent condition (609.42 ms), than in the incongruent condition (715.65 ms), being the conflict effect of 106.23 ms.

**Figure 6:**
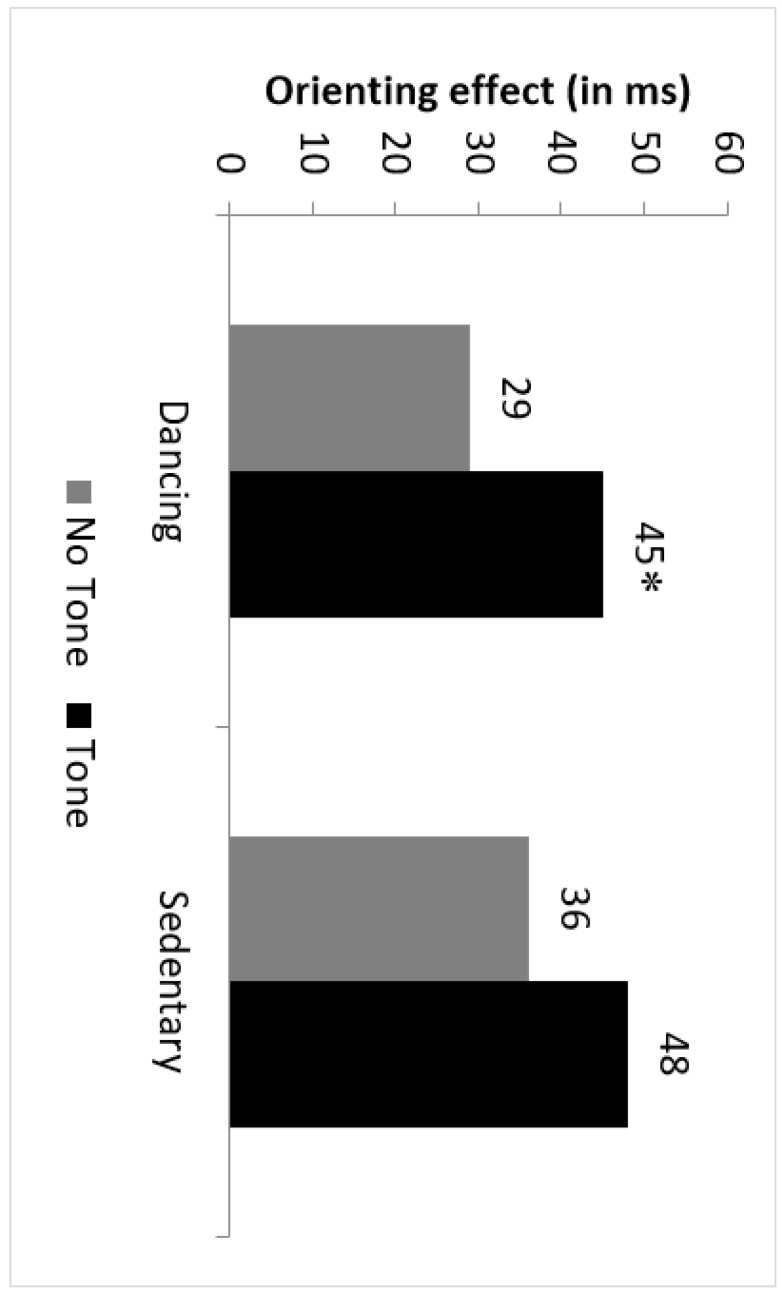
Average orienting effects (invalid RT minus valid RT) for trials with tone and non-tone (numbers over bars) in dancing and sedentary groups. The difference between both tone and non-tone trials was only significant for dancing group (* *p* < 0.05).

The Auditory Signal factor interacted with the Spatial Cue variable, F(2,50) = 7.16; *p* = 0.002; ηp2 = 0.220, because the difference between orienting effect for the trials with tone (45 ms) and for the trials without it (29 ms) was significant, F(1,25) = 4.41; *p* = 0.040; ηp2 = 0.15 (see Figure 6). Finally, we found a significant Auditory Signal x Flanker Type interaction, F(1,25) = 10.25; *p* = 0.004, ηp2 = 0.290). Post hoc analysis showed that dancing group exhibited an alerting effect when flankers were congruent with the target; irrespective of that spatial cue was valid (34 ms; F(1,25) = 29.99; *p* = 0.000; ηp2 = 0.550) or invalid (24 ms; F(1,25) = 5.47; *p* = 0.020; ηp2 = 0.180). However, when flankers and target were incongruent, the tone was advantageous only if the spatial cue informed where the target would appear (20 ms; F(1,25) = 5.71; *p* = 0.020; ηp2 = 0.190), but not when the spatial cue provided invalid spatial information, (1 ms; F(1,25) = 0.002; *p* > 0.050).

Sedentary group. The ANOVA revealed significant main effects of Auditory Signal, F(1,19) = 36.12; *p* = 0.000; ηp2 = 0.660, with faster responses in the tone (705.21 ms) than in the non-tone trials (729.38 ms), (alerting effect of 24.17 ms); Spatial Cue, F(2,38) = 21.68; *p* = 0.000; ηp2 = 0.530, showing the expected pattern (invalid 734.28 ms; valid 692.58 ms; no cue 725.02 ms), (orienting effect of 42 ms); and Flanker Type, F(1,19) = 327.40; *p* = 0.000; ηp2 = 0.950, with a conflict effect of 113 ms (incongruent 774.01 vs congruent 660.58 ms). No interactions were observed (*p* > 0.050).

#### 3.4.2. Errors

There were no significant main effects of Auditory Signal, Spatial Cue, Flanker Type, Gender or Group (all ps > 0.050). The Group and Gender factors interacted with the Alerting Network (Auditory Signal), F(1,42) = 7.03; *p* = 0.01; ηp2 = 0.14, because only women dancers made a lower percentage of error in the presence of a warning signal (3.0 %), compared to that shown in the absence of it (3.7%), F(1,11) = 4.48; *p* = 0.05; ηp2 = 0.29. This alert effect was not observed in the other groups.

The Flanker Type x Spatial Cue x Group interaction was also significant, F(2,84) = 3.59; *p* = 0.030; ηp2 = 0.08. However, this interaction was due to the inclusion of non-cue trials. Dancing group showed a percentage of error of 3% in the incongruent trials, irrespective of the spatial cue type (invalid, valid, no cue), while sedentary group exhibited a percentage of error of 2% (invalid), 1% (valid), and 3% (no cue) incongruent trials. When target and flankers were congruent, dancers made less than 1% of errors (in the invalid and no cue conditions) or none in the valid condition. Likewise, control group also made few errors (1%) in the three orienting conditions. It is worthy to note that the average percentage of error (2%) committed for both dancing and sedentary groups was very low, and no significant differences emerged between them (*p* > 0.050).

### 3.5. The Boxes Room Task

#### Errors

**Figure 7:**
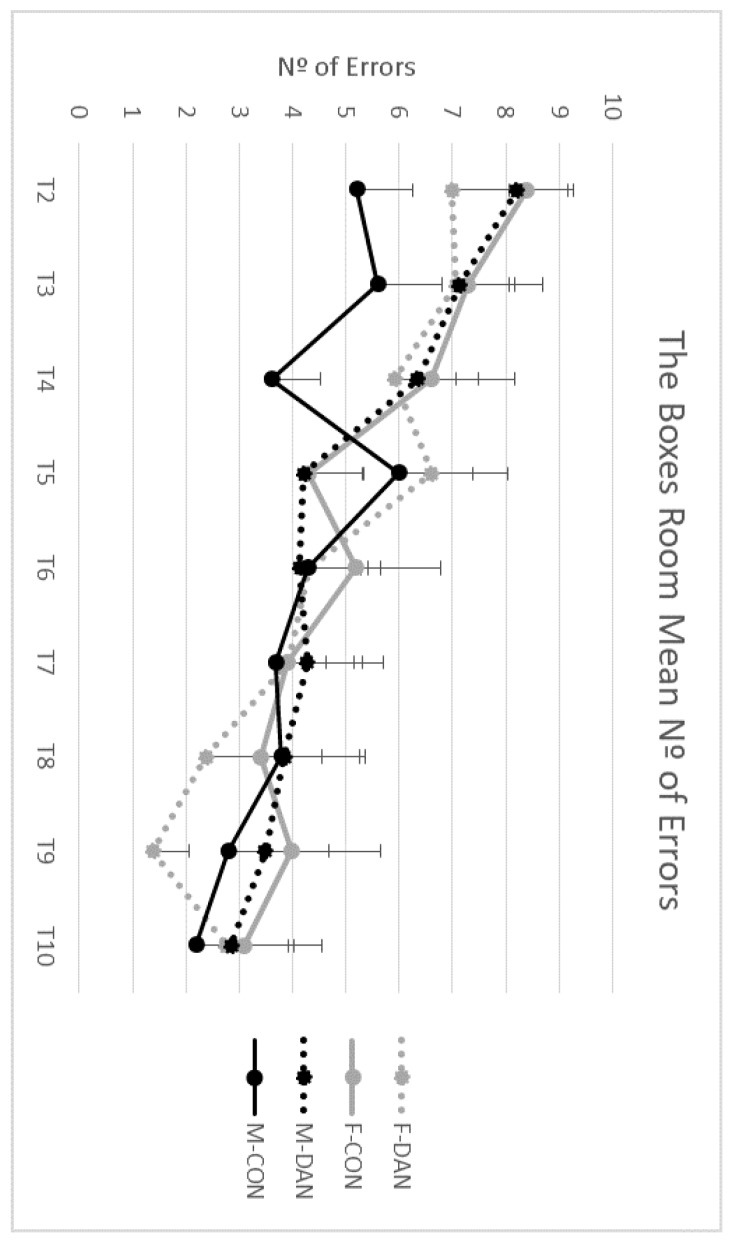
Number of errors in the spatial memory task. All groups improved across trials. Groups did not differ in the number of mistakes in the task. Male dancing (M-DAN); female dancing (F-DAN); male control (M-CON); female control (F-CON). Mean + SEM.

ANOVA showed that there was not a significant main effect of Gender, F(1,43) = 0.12; *p* = 0.720, or Group, F(1,43) = 0.02; *p* = 0.870. Nevertheless, analyses disclosed a significant main effect of Trial factor, F(8,344) = 17.44; *p* = 0.000, and a significant interaction factor Gender x Group x Trial, F(8,344) =2.50; *p* = 0.011. No significant effects were found in the interactions Gender x Trial, F(8,344) = 0.82; *p* = 0.570 or Group x Trial, F(8,344) = 0.73; *p* = 0.650. Post-hoc analyses showed that both groups (dancers and sedentary subjects) and both genders reduced their number of errors during the task, reaching the asymptotic level of performance on trial 5, but there were no differences when comparing their intergroup performance in gender and/or group (see Figure 7).

## 4. Discussion

The aim of this study was to explore the effect of dancing salsa, as an aerobic exercise, on spatial memory by using a virtual reality task, and on executive functions through the ANT-I task, in men and women. Subjects who had been practicing dancing were doing it for at least 6 months, including this activity in their normal routine. On the contrary, the control group had a sedentary lifestyle and had not practiced other sports in the last 6 months.

Regarding the scores in the neuropsychological tests, the K-BIT reported no differences in terms of intelligence between both groups, meaning that ageing-related decline was not affecting their performance. This is important because proves that the basal level was similar. Nevertheless, dancers outperformed controls in the Zoo Map test, a task involving planning ability. These results are consistent with previous studies in which dance improved executive functions [40]. Dancers are meant to have a trained observation capacity with the intention of imitating other’s moves and actions. Thus, the dorsolateral prefrontal cortex and the pre-supplementary motor area are two brain regions involved in this activity [46] as well as in the planning and generation of actions [47]. Furthermore, this better performance could be mediated by structural and functional changes in the prefrontal cortex associated to aerobic fitness. Note that practice of aerobic activities was also related to better executive functions [31,48]. Thus, a greater gray matter volume was reported in prefrontal regions [49] as well as a higher activation in prefrontal regions such as the anterior cingulate [50,51].

In the FAS test, women’s outperformed men, regardless of the group. This is not surprising if we consider that women traditionally score higher in verbal tests [52,53]. Either way, it is also important to highlight that these differences in the phonemic verbal fluency scores were milder between dancers (12.23 for women vs. 11.80 for men) in comparison with controls (11.21 for women vs. 7.5 for men). A possible explanation could be the compensation effect of dance practice. In addition, it is interesting to note that in the Animal Naming subtest, men included in the dancers group outperformed men in the control group, scoring at the same level as control women. Therefore, beyond the typical superiority of women in verbal tasks, dance practice seems to be a modulating factor that could improve performance. Thus a beneficial effect of dance was reported in a series of verbal tasks, which included the Alternative Uses test, considered together with the FAS test as a spontaneous flexibility measure [54]. Such tests disclosed differences in performance when comparing professional with novice dancers and they attributed these results to the degree of creativity of their professional dancers. It is worth noting that Age affect FAS and Animal Naming tests, as disclosed by Pearson correlations and ANCOVA test. Taking into account that men in the control group were slightly older, it is possible that these differences in verbal fluency could also be depended on age as well as in the above mentioned factors.

Regarding the ANT-I task, it should be noted that the present study follows the attentional model proposed by Posner and Petersen [19], which considerate that different attentional manifestations are produced by three attentional subsystems called the alerting network, the orienting network, and the executive control network. The three subsystems are functionally and anatomically independent neural networks, although work in cooperation to adapt information processing to the demands of the environment [30]. The alerting network provides the general activation of cortical and thalamic areas that prepare us for faster responding to any stimulus. The use of a warning signal (e.g., a tone) can induce a brain state related to alerting that differs from that activity produced by a target. The presence of a warning signal would induce a phasic extrinsic alertness related to the nonspecific activation that prepares us to response. This type of alertness is different from the so-called tonic intrinsic alertness or vigilance state, which implies sustained activation over a relatively long period of time, and varies according to the circadian rhythm (see [55], for a review about this network). The orienting network is a subsystem that allows shifting our attention around to various spatial locations in a voluntary or endogenous way, guided by our expectations of relevant locations or object features, or in an involuntary or exogenous manner as when is captured by the salient features of stimuli (e.g., an abrupt onset stimulus, form, color) (see [56], for a review). The ANT-I task used in the present study, containing trials with invalid orienting cues, allowed examining the cost of redirecting attention towards the target’s location, relative to the advantage observed when the target appears in the same location that the spatial cue (valid trial). Finally, the executive control network plays an important role for monitoring and conflict resolution, error detection, or habitual response inhibition [20]. The trials with incongruent flankers generate a situation of conflict (with the target) that participants must resolve before responding.

To the best of our knowledge, no studies explored whether salsa dancing modulates performance on this ANT-I task. Most of research has focused on studying the influence of the chronic and acute aerobic sport practice (e.g., cycling, swimming, tennis) on attentional performance. Usually, findings suggest that regular aerobic exercise produce positive effects for executive functioning in healthy young and older people (see [57], for a review). We also found the typical effects of alerting, orienting and conflict in both dancing and sedentary groups. However, dancers responded faster than non-dancers presumably due to the effect of aerobic dancing. It is interesting to note that this improvement in the speed of response is mainly due to the men who practice dance, since the women of both groups showed a similar performance pattern. Somehow, this kind of aerobic exercise seems to benefit men more than women in terms of response latency. However, hearing a tone (which would induce the start-up of the alert network) seems to positively influence the performance of the women dancers in terms of accuracy, although this was lower than that observed in the men dancers.

Also, we obtained an interesting interaction between alerting and executive networks, with a larger conflict effect in the presence of a warning signal. We had already observed this interaction using the ANT-I task in a previous study in older sportsmen [31]. According to Posner and Rothbart [58], it seems that paying attention to the tone generate a “clearing of consciousness” effect (i.e., a subjective effect of emptying thoughts) that allow us preparing to response. Thus, the alerting network would modulate (inhibiting) the executive network. Finally, the alerting network also interacted with the orienting one. The orienting effect was larger for the tone trials than non-tone trials, but only in the dancing group. The alerting network seems to act on the orienting network in a similar way as it does on the executive network in this group. Taken together, the data seem to suggest that the dancing increases the general state of alert to respond faster (especially in men dancers) or to respond more accurately (particularly in women dancers) to any stimulus that appear in the visual field.

On the other hand, the Boxes Room Task has proven to be an effective test to assess spatial ability over traditional neuropsychological test [59]. This task usually discloses a gender dimorphism effect [60,61]. This was not the case in our study which reported that both men and women delivered a similar performance. Nevertheless, these results agree with another study which used the same paradigm with similar age groups, thus reporting no gender differences in the middle age group 45–54, and older adults 55–64 [62]. This could be due to the fact that the level of difficulty in this task was not adjusted correctly. Hence, when the task is too easy the dimorphic effect does not appear. Accordingly, it is necessary to find an optimal level of difficulty [8]. Our results also confirm recent findings reporting that women are as accurate as men [63] provided that both groups are given a sufficient amount of time to learn spatial relationships and repetitions. Therefore, the degree of familiarity with the environment would play a role as a modulating factor, making up for men’s advantage. Another aspect to consider is the heterogeneity of the sample. The ages ranged from 49 to 70 years old, a quite high variability that might have added important inter-individual differences in our sample.

In addition, it is well known that the hippocampus plays an important role when performing the Boxes Room task [64]. It has been demonstrated that physical activity enhances the functioning of the hippocampus and other brain structures involved in spatial orientation. Thus, some studies suggest that physical activity is associated with a larger volume of the hippocampus [65] and hippocampal integrity [66], and consequently an aged-related loss of the hippocampal volume due to age can be compensated through the practice of aerobic sports [36].

Furthermore, the construct of cognitive reserve has been commonly related to healthy ageing, and its assessment includes different educational and recreational brain-stimulating activities across the lifetime [67]. A larger cognitive reserve is associated with a delaying in the onset of dementia and normal cognitive impairments in elderly [68]. Considering this, a better cognitive functions would be expected for subjects included in the dancer group in comparison with those in the control group, since dancing, as a physical exercise, is included among the factors that contribute to cognitive reserve [69]. However, sample heterogeneity, as mentioned before, might impede clearer differences since this neuroprotective effect of cognitive reserve would be more evident in aged participants, 60–70 years old, when cognitive decline becomes more pronounced [31].

Regarding the time needed for these changes in the brain to manifest, all the participants who took part in this study had been dancing regularly for a minimum of 6 months. However, in most of them this period was way longer, 8 years on average. Previous studies demonstrated that this period is enough for the benefits of sport practice to have an effect in the brain. In a study carried out by Erickson et al. [36] the hippocampus increased its volume after a 6-month exercise intervention. Maass, Duzzel, Goerke, Becke, Sobieray, and Neumann et al. [70] also reported an evident beneficial effect of aerobic exercise even after shorter interventions (3 months), since participants experienced a hippocampal volume increase and substantial memory improvements. Other studies obtained similar results in patients with depression [71] and schizophrenia [72] after a 3-month intervention. In addition, procedural and motor learning, such as dancing, entails a plastic reorganization of the human brain through functional and structural changes. Interestingly, our brain changes not only when we perform motor sequences, but also when we imagine practicing these exercises [73,74]. It would be interesting to determine the effect of dancing programs through virtual environments or mental practice in individuals with motor difficulties.

Finally, the interpretation of our results should take into account the following limitations. Thus, ours is a quasi-experimental study and participants were not randomly assigned to the groups. It is also important to note that other healthy habits that could be influencing cognitive functioning (e.g., diet, sleep routine) were not considered, and therefore it is impossible to determine to what extend they could influence on these results. Another important limitation is the sample’s heterogeneity, since the age range was quite large. Increasing the sample and clustering participants by ages could solve this problem. Nevertheless, the number of participants is similar to previous studies using virtual reality-based tasks [75]. Thus, 10–15 participants per group seems to be a correct number to achieve valid conclusions. In our case, previous experiments with similar tasks provided replicable data [8,61]. Also dancing involves many different activities, including social contact, learning rhythms, and motor coordination which could be reflected on tests scores. It is difficult to separate the weight of each variable. More important, the general fitness level was not assessed and it is impossible to determine how this variable contributes to performance. Measures like VO2max could be included in further studies.

## 5. Conclusions

This study represents a first approach to determine the cognitive effects of dancing. The results proved that dancing have a beneficial effect in executive and more limited on spatial memory functions. This study suggests that cognitive stimulation therapies based on dancing could help to maintain or even improve cognitive skills, reducing age-related decline. Future research on this topic should consider an experimental design with bigger samples including not only healthy people but also persons with mild cognitive impairment.

## Figures and Tables

**Table 1:** Characteristics of the sample (mean + SD).

	Dancers				Sedentary			
	Men		Women		Men		Women	
	Mean	SD	Mean	SD	Mean	SD	Mean	SD
Age	57.1	5.8	54.9	4.35	61	5.1	54.2	3.5
Educational Level 1–3	2	0.78	2.33	0.77	2	0.9	1.9	0.9
Years Dancing	9.1	7.9	7.4	7.8	-	-	-	-
Videogame Experience 1–4	1.35	0.6	1.58	0.9	1.1	0.3	1.7	1.1

**Table 2:** Mean RT (in milliseconds), and standard deviations (in italics) for each experimental condition of alerting (no tone, tone), orienting (invalid, no cue, valid), and conflict (congruent, incongruent) in the modified ANT-I task.

	Congruent		Incongruent	
	Dancers	Sedentary	Dancers	Sedentary
No Tone				
Invalid	634	684	736	797
	*21*	*24*	*19*	*22*
No Cue	637	694	736	791
	*19*	*22*	*19*	*22*
Valid	610	649	702	761
	*18*	*21*	*19*	*21*
Tone				
Invalid	613	671	735	785
	*18*	*21*	*19*	*22*
No Cue	587	644	703	772
	*18*	*20*	*19*	*21*
Valid	576	622	682	738
	*18*	*21*	*20*	*22*
